# Recent Advances in Biomarkers for Parkinson’s Disease

**DOI:** 10.3389/fnagi.2018.00305

**Published:** 2018-10-11

**Authors:** Runcheng He, Xinxiang Yan, Jifeng Guo, Qian Xu, Beisha Tang, Qiying Sun

**Affiliations:** ^1^Department of Neurology, Xiangya Hospital, Central South University, Changsha, China; ^2^National Clinical Research Center for Geriatric Disorders, Changsha, China; ^3^Key Laboratory of Hunan Province in Neurodegenerative Disorders, Central South University, Changsha, China; ^4^Center for Medical Genetics, School of Life Sciences, Central South University, Changsha, China; ^5^Parkinson’s Disease Center of Beijing Institute for Brain Disorders, Beijing, China; ^6^Collaborative Innovation Center for Brain Science, Shanghai, China; ^7^Collaborative Innovation Center for Genetics and Development, Shanghai, China; ^8^Department of Geriatrics, Xiangya Hospital, Central South University, Changsha, China

**Keywords:** Parkinson’s disease, biomarkers, biochemical, neuroimaging, genetics

## Abstract

Parkinson’s disease (PD) is one of the common progressive neurodegenerative disorders with several motor and non-motor symptoms. Most of the motor symptoms may appear at a late stage where most of the dopaminergic neurons have been already damaged. In order to provide better clinical intervention and treatment at the onset of disease, it is imperative to find accurate biomarkers for early diagnosis, including prodromal diagnosis and preclinical diagnosis. At the same time, these reliable biomarkers can also be utilized to monitor the progress of the disease. In this review article, we will discuss recent advances in the development of PD biomarkers from different aspects, including clinical, biochemical, neuroimaging and genetic aspects. Although various biomarkers for PD have been developed so far, their specificity and sensitivity are not ideal when applied individually. So, the combination of multimodal biomarkers will greatly improve the diagnostic accuracy and facilitate the implementation of personalized medicine.

## Background

Parkinson’s disease (PD) has become the second most common neurodegenerative disease following Alzheimer’s disease (AD) and is estimated to occur in about 1% of the population over the age of 60 and 4% of the individuals aged over 80 years (Lindqvist et al., 2013; Elbaz et al., 2016). The major pathological changes in PD patients are the progressive degeneration of dopaminergic neurons in the substantia nigra and the accumulation of intraneuronal inclusions of the α-synuclein, which are called Lewy bodies (Jellinger, 2012). Clinically, PD mainly manifests as motor symptoms, such as bradykinesia, resting tremor, muscle rigidity and postural instability. PD also appears to be correlated with nonmotor symptoms, including olfactory dysfunction, sleep problems, constipation, depression and dysautonomia, due to the neuronal loss in several other brain areas that may occur before or after dopaminergic neurons are lost. The diagnosis of PD in living patients is now mainly based on clinical manifestations, according to the United Kingdom Brain Bank Criteria and the MDS Clinical Diagnostic Criteria (Rodriguez-Oroz et al., 2009; Mehta and Adler, 2016). However, PD shares many common clinical features with some other neurodegenerative diseases, including the synucleinopathies multiple system atrophy (MSA), progressive supranuclear palsy (PSP), dementia with Lewy bodies (DLB), corticobasal degeneration (CBD), etc., making its diagnosis complicated. It has been reported that the misdiagnosis rate can be as high as 25% in early stage of the PD (Meara et al., 1999). Therefore, reliable biomarkers are necessary for the early and accurate diagnosis to predict the disease occurrence and progression, as they can be used as objective and characteristic evaluation indicators of normal biological processes and pathogenic processes of the disease (Robb et al., 2016).

PD is a complex and progressive neurodegenerative disease whose progress course can be divided into three stages: including preclinical PD stage with neurodegenerative processes already commenced but without evident symptoms or signs; prodromal PD stage, in which symptoms and signs have been present, but they are yet inadequate to define this disease; and clinical PD stage, where diagnosis is based on the presence of classical motor features (Ferrer et al., 2012; Olanow and Obeso, 2012; Sharma et al., 2013). The development of appropriate biomarkers that facilitate the early diagnosis, detecting disease progression and the discovery of new treatments for PD are crucial for better clinical management of PD patients. As a result, the establishment of reliable biomarkers will become an advanced subject of extensive investigation. In this review article, we will explore the recent advances in PD biomarker research for disease diagnosis and disease surveillance from a variety of clinical, biochemical, genetic and neuroimaging perspectives.

## Clinical Biomarkers

The motor symptoms of bradykinesia, resting tremor and muscle rigidity can be considered as the most significant and direct diagnostic marker for PD. At the same time, these motor features can also be utilized to monitor response to medical treatments and evaluate disease progression in PD. For the early diagnosis of the disease, many non-motor features, including hyposmia, rapid eye movement (REM) sleep behavior disorder, and constipation, except for the motor features, which can occur during all stages of the PD and may even be highly observed a few years before motor symptoms onset, are receiving increasing attention as they may be helpful in the detection of prodromal PD.

### Olfactory Dysfunction

As early as in 1975, Ansari and Johnson had proposed the relationship between olfactory dysfunction and the incident of PD (Ansari and Johnson, 1975). Also, a large-scale longitudinal study of 2,263 elderly men aged 71–95 years evaluated the sense of smell using a 12-odor smell identification test by Ross et al. (2008) and confirmed that olfactory dysfunction is related to the development of PD in the 8-year follow-up. Subsequently, many studies reported that roughly 75% of PD patients have a raised odor detection threshold, and as high as 90% of them suffer from odor identification deficits (Doty et al., 1988; Doty, 2007). Many other studies also showed that olfactory dysfunction in asymptomatic first-degree relatives of PD is associated with elevated risk to develop clinical PD (Berendse and Ponsen, 2006). With the current advances in pathology and brain imaging analysis techniques, it has been revealed that neurodegenerative changes, including the existence of abnormal cytoplasmic proteinaceous inclusions (Lewy bodies), are commonly observed in brain areas responsible for the olfaction, such as the olfactory bulb, hippocampus, amygdala and orbitofrontal cortex (Silveira-Moriyama et al., 2009; Funabe et al., 2013; Khoo et al., 2013; Saito et al., 2016). Therefore, the olfactory system has been considered to be the origin of PD pathology (Khoo et al., 2013). According to Braak staging theory, the pathological process progresses of PD are divided into six stages. This theory proposed an upward course in the pathology of the brain, beginning in the olfactory bulb, and the motor nucleus X of the dorsal nucleus, then reaching the lower brainstem and eventually stretching into the cerebral cortex (Braak et al., 2003; Goedert et al., 2013). These results suggest that olfactory dysfunction is more frequently observed than motor symptoms in PD, and the olfactory impairment may precede the motor symptoms by a few years (Goldman and Postuma, 2014). Consequently, olfactory dysfunction can be recognized as an early diagnostic marker of PD (Hoyles and Sharma, 2013). Olfactory dysfunction as one of the latest PD diagnostic criteria for MDS 2015, the diagnostic specificity of PD can be as high as 80% (Postuma et al., 2015a). Currently, the UPSIT and Sniffin’s Sticks are the most widely used instrument to test olfactory identification. In particular, Sniffin’ Sticks can test odor detection threshold, odor discrimination, and odor identification by using a pen-like device (Casjens et al., 2013). Additionally, it is also reported that hyposmia is associated with impaired memory and hyposmia is a significant clinical marker for cognitive dysfunction in PD (Baba et al., 2012; Kawasaki et al., 2016). Even so, the utility of hyposmia as an independent predictive diagnostic method for PD is limited because olfactory dysfunction can also be detected in other diseases, such as AD and idiopathic REM sleep behavior disorder (iRBD), although it appears to distinguish PD from vascular parkinsonism, MSA and CBD (Doty et al., 1991; Huang et al., 2016; Krismer et al., 2017). Therefore, combining with other non-motor features in PD such as RBD, depression, constipation and visual symptoms, the specificity of olfactory dysfunction as a biomarker for PD can be significantly enhanced (Berg, 2012).

### Rapid Eye Movement Sleep Behavior Disorder (RBD)

RBD is a parasomnia effecting REM sleep characterized by the occurrence of atypical motor or cognitive events during REM sleep due to the reduction of the normal skeletal muscle atonia, thus leading to abnormal nocturnal behavior, such as punching, kicking, flailing and vocalization, self-inflicted injuries or injuries of bed partners (Dauvilliers et al., 2013). Up to date, RBD has been supposed to be the most common and best-characterized parasomnia in PD with an approximated prevalence between 30% and 59% (Ylikoski et al., 2014). At the same time, imaging studies in RBD patients reveal a little but a quite significant symmetrical loss in striatal dopaminergic uptake. This result reflects the neuronal loss and α-synuclein degeneration of the brainstem nucleus, so the brain region affected by RBD is in the second phase of the Braak hypothesis (Braak et al., 2003), RBD maybe precede the emergence of other symptoms of PD in the Braak disease flow chart, and it can become suggestive of preclinical PD.

The evidence is very clear that RBD is correlated with neurodegenerative diseases, especially with the α-synucleinopathies, including PD, MSA and DLB et cetera (Nofzinger, 2010; Oertel et al., 2014). RBD can precede the clinical symptoms of these diseases by several years, and the risk rate of the these diseases is between 30% and 91% after being diagnosed with RBD (Mahowald and Schenck, 2013; Iranzo et al., 2014; Postuma et al., 2015b). Therefore, RBD is assumed to be an original symptom of progressive neurodegeneration. In addition, imaging of presynaptic dopaminergic function shows a decrease in striatum dopamine levels in RBD patients, consistent with the abnormal metabolism of the network level observed in PD patients (Ellmore et al., 2013). As a result, RBD as a preclinical marker for PD may play an essential role in identifying an at-risk cohort of individuals who maybe go on to develop PD in the future. Furthermore, the presence of RBD is closely related to the dementia. PD patients with cognitive dysfunction have a further reduction in EEG activity and performance after awakening, which can also be observed in RBD patients without cognitive impairment (Bang et al., 2016). Thus, RBD may be a potential biological biomarker for the development of PD symptoms into dementia. Generally, RBD may be a suitable clinical biomarker for diagnosis of prodromal PD particularly when utilized in combination with other biomarkers; and polysomnography (PSG) is the gold standard for RBD diagnosis (Medicine AAOS, 2014).

### Constipation

α-synuclein degeneration of the autonomic nervous system in PD also contains the myenteric plexus denervation of the colonic sympathetic. Therefore, constipation is the most common symptom of autonomic dysfunction in PD patients. The Rome criteria is an internationally accepted set of objective findings that used for defining functional constipation. It focuses on six features: hard and dry stools, straining, incomplete evacuation, the sensation of anorectal obstruction or blockage, two or fewer bowel movements (BMs) per week, and manual maneuvers to procure evacuation (Drossman et al., 2010). According to the Rome criteria, several studies indicated that constipation may occur prior to the onset of motor symptoms in PD (Cersosimo et al., 2013). The most convincing evidence comes from the Honolulu Heart Program, which is a long-term prospective cohort survey of 8,006 Japanese American men enrolled. This study suggested that PD incidence will increase obviously with decreasing BMs per day, when participants without PD are prospectively followed up (Petrovitch et al., 2009 ). Additionally, this study also demonstrated that Incidental Lewy Body (ILB) pathology can be present in almost 25% of participants with constipation compared with 6.5% of participants without it (Ross et al., 2012). All these results appeared to be consistent with the PD pathological staging system raised by Braak et al. (2003), who has proposed that early neuropathological changes of PD are present in the enteric autonomic nervous system, especially the dorsal nucleus of the vagus nerve. Consequently, constipation may be thought to have the potential sensitivity to reflect the prodromal phase of PD. Another retrospective study also reported that the presence of α-synuclein in colonic biopsies can take a few years prior to the occurrence of motor symptoms in PD (Cersosimo and Benarroch, 2012). Furthermore, histological analysis of enteric nervous system (ENS) by colonoscopy biopsy may play an important role in pathological diagnosis of preclinical PD.

In addition, PD patients also have several other non-motor symptoms, including depression, symptomatic hypotension, and sexual dysfunction. Although the predictive value of a single symptom is low, and when multiple symptoms occur simultaneously there will be an utmost high predictive value.

## Biochemical Biomarkers

Accurate and early diagnoses are significant for a successful treatment in PD patients; and sensitive and selective biochemical biomarkers will play an essential role in the detection of prodromal PD. As we all know, biochemical biomarkers can be investigated both in body fluids and tissues. Several potential biochemical biomarkers have been already found in blood, saliva, cerebrospinal fluid (CSF) and biopsies. But for now, a combination of multiple biomarkers may be crucial to achieve high sensitivity and specificity of diagnosis. At present, an increasing number of experiments have demonstrated that mitochondrial dysfunction, oxidative stress, Lewy bodies formation, neuroinflammation and other mechanisms involved in the development of PD. So, we will review the biochemical biomarkers from the above aspects.

### Mitochondrial Dysfunction and Oxidative Stress Related Biomarkers

#### Urate

Urate, generated in the liver and the small intestine, plays a potent antioxidant role by removing singlet oxygen, hydroxyl radicals, hydroxyl peroxide and peroxynitrite. A lack of antioxidative capabilities may make cells more vulnerable to free radicals. The antioxidant defense of urate has been considered to be particularly important for preventing oxidative damage in the human brain. Oxidative stress may be one of the cause of the loss and degeneration of dopaminergic neurons in the substantia nigra of PD (Hwang, 2013). Although the biological mechanisms that lead to PD has not yet been completely discovered, several studies showed that they are likely to include oxidative stress-induced damage (Perfeito et al., 2012). As a result, urate is likely to be protective against PD, as supported by several experiments. *in vitro*, urate can prevent spontaneous degeneration of cultured substantia nigral neurons in the PD models, as well as the death of dopaminergic neurons caused by oxidative and mitochondrial toxins (Cipriani et al., 2012a,b). Meanwhile, *in vivo*, in the PD mouse models, elevated concentrations of urate in the central nervous system (CNS) results in the improved phenotype and histopathologic findings (Gong et al., 2012; Chen et al., 2013). Two clinical trials, Deprenyl and Tocopherol Antioxidative Therapy of Parkinsonism (DATATOP) and Parkinson Research Examination of CEP-1347 Trial (PRECEPT), all suggested that higher level of serum urate is closely related to a slower rate of disease progression (Ascherio et al., 2009). This study not only established correlation between serum urate levels and clinical progression of PD, but also identified CSF urate concentration as a predictor of rate of clinical reduce in PD for the first time. These findings facilitated a phase II clinical experiments testing urate increase as a treatment strategy for disease modification. In a secondary end point in the PRECEPT trial, the unified PD rating scale (UPDRS) score of PD patients with lower blood urate levels was obviously higher than the control group (Schwarzschild et al., 2008). The concentration of urate in CSF, which accounts for about 10% of peripheral blood concentration, depends on the concentration of urate in the peripheral blood and the integrity of the blood-brain barrier. A recent study carried by Ascherio et al. (2009) demonstrated a negative association between CSF urate levels and subsequent clinical incidence of PD and the rate of change in the UPDRS. A convergence of data identified that urate in serum and CSF, as an protective factor for PD, are robust biomarkers of PD. Additionally, DATATOP Trial also found that patients with cognitive dysfunction have obviously lower urate concentrations compared with PD patients without cognitive dysfunction (Gunzler et al., 2011); and a Chinese study confirmed a negative correlation between urate levels and severity of cognitive dysfunction in PD as well (Wang et al., 2009). In general, urate can be used as a promising marker for diagnosing PD and detecting disease progression.

#### Protein DJ-1

Protein DJ-1 is an ubiquitous multifunctional protein that participates in so many cellular metabolic processes, including neuroprotective role in oxidative stress during neurodegeneration, RNA binding, chaperone, protease and mitochondrial regulation (Saito, 2014). Diminished function of DJ-1 may lead to the occurrence of oxidative stress-related diseases, such as PD. So mutations in the *DJ-1* gene PARK7 are related to inherited recessive PD (Abou-Sleiman et al., 2010). Several studies showed that DJ-1 levels are elevated in CSF or plasma of PD patients compared to non-PD controls (Waragai et al., 2010). Meanwhile, Waragai et al. (2007) suggested that there is a positive correlation between DJ-1 levels and the disease stage assessed by H&Y scores. On the other hand, several experiments contradicted these conclusions. The discrepancy of these results may result from the methodological variations, including discrepancies in techniques or sample sizes, and the contamination of CSF by blood cell containing a higher concentration of DJ-1 (Shi et al., 2010; Hong et al., 2010). So, when researchers used quantitative Western blotting and a highly sensitive newly developed Luminex assay examining the CSF DJ-1 levels without the contamination of blood, they demonstrated that DJ-1 levels in CSF were decreased in PD patients compared with normal controls, AD patients and MSA patients by several experiments. But there is no association between DJ-1 levels and the severity of PD (Shi et al., 2011; Herbert et al., 2014). In spite of this, the DJ-1 levels may be an important candidate for a PD biomarker. Moreover, extensive experiments suggested that the cysteine residue at position 106 (Cys-106) is preferentially oxidized when it is exposed to oxidative stress. The antioxidative action of the Cys-106 determines the functions of DJ-1 (Saito, 2014). Moreover, the levels of DJ-1 oxidation at Cys-106 in the erythrocytes of untreated PD patients are markedly elevated when compared with medicated patients and non-PD controls (Saito et al., 2009). So the Cys-106 oxidative response plays an important role in PD pathogenesis (Hao et al., 2010; Ariga et al., 2013; Saito and Noguchi, 2016).

Additionally, Lin et al. (2012) have showed that the level of blood DJ-1 modified by 4-hydroxy-2-nonenal (4-HNE) is significantly decreased in late-stage PD patients, suggesting that 4-HNE modified DJ-1 may potentially be one of the biomarkers of disease severity in the body fluid. The oxidized DJ-1 is promising to be a useful biomarker (Lin et al., 2012).

#### Coenzyme Q10

Reactive oxygen species (ROS) make great contribution to the pathophysiology of PD. Coenzyme Q10 (CoQ10) is an antioxidant that facilitates function of the mitochondrial electron transport chain (Kikusato et al., 2016). *In vivo* and *in vitro* trials showed that the defect in the activity of mitochondrial complex 1 in PD patients will lead to the disruption of redox equilibrium and neuronal toxicity. As a lipophilic antioxidant, CoQ10 is capable of preventing neurodegeneration against mitochondrial deficiency. As we all know, ubiquinol-10 (reduced form of coenzyme Q10, CoQH2-10) has been oxidized into ubiquinone-10 (oxidized form of coenzyme Q10, CoQ-10) at the early stage of oxidation in the human plasma, so the percentage of oxidized form in total coenzyme Q10 (%CoQ-10) will be a useful biomarker of oxidative stress (Yen et al., 2014). Sohmiya et al. (2004) found a significant decrease in plasma levels of total CoQ10 and an obviously increase in plasma %CoQ-10 in PD patients compared to non-PD controls. Additionally, %CoQ-10 is likely to increase with the disease progress evaluated by H&Y scores (Sohmiya et al., 2004). However, there is no meaningful discrepancy in total CoQ-10 level or %CoQ-10 between AD patients and normal controls (Sohmiya et al., 2004). It is suggested that levels of CoQ10 and %CoQ-10 have potential as biomarkers in the diagnosis of PD. Additionally, supplementation of CoQ10 may be a promising means of prevention and treatment on PD.

#### Homocysteine (Hcy)

Homocysteine (Hcy) is a natural amino acid generated by the body’s methylation process. It is well known that hyperhomocysteinemia has become an important risk factor for cerebrovascular diseases in the general population (Leng et al., 2017). On the other hand, the toxic effects of Hcy on dopaminergic neurons have been confirmed *in vitro* and *in vivo* experiments (Kocer et al., 2016). Hyperhomocysteinemia will be neurotoxic to the substantia nigra by damaging neuronal DNA and stimulating NMDA receptors, contributing to accelerated death of dopaminergic neurons. In addition, increased Hcy levels will lead to the exacerbation of oxidative stress in dopaminergic neurons, causing the MPTP-induced PD-like pathology (Obeid et al., 2009). Therefore, hyperhomocysteinemia is likely to be correlated with dyskinesia and it will become an indicator of neurodegeneration (Zoccolella et al., 2006). The Hcy levels in patients with PD, MCI, AD and cerebral amyloid angiopathy (CAA) are relatively higher than normal controls, but PD patients have the highest levels compare to the other patients (Irizarry et al., 2005). Increased concentrations of Hcy in the CSF and plasma are supposed to be risk factors for PD (Rozycka et al., 2013). Furthermore, higher Hcy levels are considered to be correlated with worse cognition and higher plasma amyloid beta (Aβ) levels in PD patients (Irizarry et al., 2005; Zoccolella et al., 2006). Although the acute levodopa therapy will increase plasma Hcy levels, folate and vitamin B12 are very effective to reverse the levodopa-related hyperhomocysteinemia (Postuma et al., 2006).

#### 8-Hydroxydeoxyguanosine (8-OHdG)

8-Hydroxydeoxyguanosine (8-OHdG) is the main product of oxidation reaction of hydroxyl radicals and guanine residues in DNA and is one of the best biomarkers of DNA damage due to oxidative stress. Several studies implicated that 8-OHdG concentrations are elevated selectively in the substantia nigra of PD patients (Alam et al., 1997). Consequently, we speculate that oxidative DNA damage plays a significant role in the pathogenesis of PD. Therefore, the 8-OHdG levels in the CSF and serum of PD patients are significantly higher than normal controls (Gmitterová et al., 2009; Isobe et al., 2010; García-Moreno et al., 2013). Furthermore, the concentrations of 8-OHdG are positively associated with the duration of illness and %CoQ-10 (Isobe et al., 2010). It is well known that the excised 8-OHdG is excreted in urine, so the increased urinary 8-OHdG concentrations in PD patients are much higher than control subjects as well. The urinary 8-OHdG concentrations are also related with Hoehn-Yahr stage. In addition, urinary 8-OHdG concentrations correlate most with hallucinations with the correlation coefficient of 0.857 (Hirayama et al., 2011). Therefore, the 8-OHdG, especially the urinary 8-OHdG, is likely to be a reliable biomarker for the diagnosis of PD.

#### Advanced Oxidized Protein Products (AOPP)

Advanced oxidized protein products (AOPP) is a reliable biomarker of halogenative stress, and protein halogenation is a kind of oxidative stress induced by phagocytic overstimulation. When quantifying AOPP concentrations with enzyme-linked immunosorbent assays (ELISA), researchers find that AOPP levels in serum of PD patients are significantly higher than control subjects. But there is no significant difference in CSF AOPP levels, because basal CSF levels are very low in all individuals. In addition, it has been demonstrated that the higher the Hoehn-Yahr stage or levodopa dose in PD patients or the longer the disease duration, the lower the serum AOPP levels (García-Moreno et al., 2013). So AOPP will play an important role in diagnosis of PD and monitoring disease progression.

### Abnormal Protein Accumulation and Aggregation Related Biomarkers

#### α-synuclein

The α-synuclein consisting of 140 amino acids expresses mainly in neurons, more particularly in neuronal synapses. Although the physiological role of the α-synuclein is not yet completely understood, it seems that the regulation of synaptic plasticity is one of its roles. As we all know, the presence of Lewy bodies, which are protein aggregates with α-synuclein in surviving neurons, is one of the pathological features of PD. While the α-synuclein is the main component of Lewy bodies cytoplasmic inclusions (Bandopadhyay, 2016). The phosphorylation, misfolding and abnormal accumulation of α-synuclein play a significant role in the pathogenesis of PD. In addition, α-synuclein is the production of the *SNCA* gene; its mutation can cause one of the monogenetic forms of PD. So Braak’s theory of spreading neuropathology leads to the hypothesis of prion-like spread of misfolded α-synuclein (George et al., 2013). Histological studies measured the levels of CSF α-synuclein with ELISA and concluded that the amount of CSF α-synuclein in PD patients is significantly lower than non-PD controls (Mollenhauer et al., 2011; Park et al., 2011; Parnetti et al., 2014). Furthermore, a large degree of negative correlation between CSF α-synuclein levels and Hoenh-Yahr stage reflecting disease severity has been found (Tokuda et al., 2006). Real-time quaking-induced conversion (RT-QuIC) provided a novel method for the detection of abnormal α-synuclein by using aggregated α-synuclein to induce further aggregation of non-aggregated α-synuclein in a cyclic manner. It can detect abnormal CSF α-synuclein in PD patients with a sensitivity of 95% and a specificity of 100% (Fairfoul et al., 2016). Groveman and his colleagues demonstrated that RT-QuIC assay can be completed within just 1–2 days by exploiting a mutant recombinant α-synuclein substrate under optimized reaction conditions, so this assay is suitable for the detection of prodromal PD due to its rapid detection capability and high diagnostic accuracy (Groveman et al., 2018). In addition, several studies suggested that the soluble oligomeric α-synuclein formed during the α-synuclein fibrosis is the most neurotoxic specie. The toxicity of it appears to be associated with the establishment of aberrant protein interactions, the interference with the protein clearance mechanisms, and the impairment of biomembranes (Gallea and Celej, 2014). As a result, a significant increase in oligomeric forms of α-synuclein in PD have been showed by Tokuda et al. (2011). Calculating the ratio of oligomeric α-synuclein (o-α-syn)/total α-synuclein (t-α-syn) can distinguish PD from controls with a specificity of 90.6% and sensitivity of 89.3% with an area under the curve (AUC) of 0.948 (Tokuda et al., 2011). However, several studies performed by different groups showed the conflictive conclusions about the blood α-synuclein levels in PD patients compared with non-PD controls (Duran et al., 2010; Prakash and Tan, 2010). The variability of these results may be due to the inconsistent methods of the sample collection and treatment, and the release of a large number of α-synuclein by erythrocyte rupture. Even so, El-Agnaf et al. (2006) suggested that oligomeric forms of α-synuclein in blood of PD are significantly more than those of non-PD controls by using an ELISA that only monitors oligomeric “soluble aggregates” levels of α-synuclein with a specificity of 85.2% and sensitivity of 52.9%. However, the early diagnosis of PD is limited by the lack of sensitive. Currently, it has been reported that the ratio of RBC oligomeric α-synuclein/total protein in PD patients is significantly higher than that in normal controls. So the ratio of α-synuclein oligomer/total protein is an effective way to distinguish PD patients from normal, with a sensitivity of 79.0% and a specificity of 64.7% (Wang et al., 2015).

The phosphorylated α-synuclein concentrations in PD patients are significantly higher than in healthy controls as well. However, Foulds et al. find that the levels of α-synuclein in plasma vary largely between individuals so the most promising diagnostic assay may be for phosphorylated α-synuclein (Foulds et al., 2013). We can use phosphorylated α-synuclein for a biomarker in PD patients. Phosphorylated α-synuclein is the most common posttranslational protein modification of α-synuclein. Several studies have demonstrated that the phosphorylated α-synuclein result in the death of neurons (Volpicellidaley et al., 2011). As the disease progresses in PD, the levels of soluble and non-phosphorylated α-synuclein reduce in vulnerable brain regions and the protein becomes increasingly more phosphorylated and insoluble (Foulds et al., 2011). Phosphorylated α-synuclein will more accurately reflect the fundamental neuropathology of PD. It can inhibit α-synuclein fibrillization and it often occurs after Lewy bodies formation. This would demonstrate that α-synuclein phosphorylation occurs relatively late in the process of the disease. Consequently, it would be a more useful biomarker for disease progression in PD and it may be essential in future drug trials, especially for disease-modifying agents to slow the progression of disease (Foulds et al., 2013). Moreover, the CSF phosphorylated α-synuclein levels are significantly higher than normal, MSA and PSP patients (Wang et al., 2012). So, we could identify PD and MSA, or PSP patients based on CSF phosphorylated of α-synuclein levels.

Recent studies suggest that the injection of short α-synuclein fibrils can promote chemokines and major histocompatibility complex II (MHCII) induction in microglia, and peripheral macrophage and monocyte recruitment. MHCII induction persists over time and spreads to other parts of the brain (including striatal) at 6-months post-injection concomitant with inclusion spread, ultimately leading to the progressive dopaminergic neurodegeneration (Abdelmotilib et al., 2017; Harms et al., 2017). These results suggest that α-synuclein fibrils are potentially involved in the early stages of PD progression and α-synuclein fibrils are promising to become candidate biomarkers for the preclinical PD.

Furthermore, α-synuclein can be cleaved into a series of aggregation-prone truncated species by plasmin, calpain 1, neurosin and 20S proteasome. C-terminally truncated α-synuclein fibrils tend to be shorter than fibrils, and were bundled laterally, while N-terminally truncated α-synuclein formed long fibrils. Both C-terminal and N-terminal truncations of α-synuclein induce fibril polymorphs and increase the cross-seeding *in vivo*. In addition to these, N-Terminal 10-/30-residue truncated α-synuclein fibrils can change the fibril conformation and decrease fibril stability, promoting α-synuclein fibrils to propagate through a prion-like molecular mechanism; and C-terminally 20-residue truncated fibrils can induce distinct structures of α-synuclein aggregates and affect their prion-like pathogenicity, leading to phenotypic diversity. These studies concluded that the α-synuclein truncation levels can be used for the diagnosis of PD. Overall, combining these indicators with other markers will provide more accurate information.

Beyond that, α-synuclein pathogenic forms may also occur in Lewy body precursors of the submandibular gland of PD patients. Recently, Adler et al. (2014) also used salivary gland tissue needle core biopsies to detect Lewy bodies and 75% of PD patients can be tested positive. Therefore, the abnormal α-synuclein expression in submandibular glands can be recognized as a new potential biomarker of preclinical PD as well.

#### Ubiquitin C-Terminal Hydrolase-L1 (UCH-L1) and β-Glucocerebrosidase

Ubiquitin C-terminal hydrolase-L1 (UCH-L1) was originally discovered as one of the brain-specific proteins by Jackson and Thompson (1981). It plays a critical role in regulating brain protein metabolism, by coupling to the proteasome pathway to remove the excessive, oxidized or other misfolded and modified proteins in neuronal cytosol (Betarbet et al., 2005; Bishop et al., 2016). So, the impairment of function or quantity of UCH-L1 will result in the reduced degradation rate of α-synuclein. So that it will lead to the aggregation of intracellular Lewy bodies, eventually the neurodegeneration and neurocyte death. The UCH-L1 concentrations in CSF of PD patients have been demonstrated to be decreased compared to normal controls (Jiménez-Jiménez et al., 2014). Particularly, the PD patients present the lowest levels of CSF UCH-L1 differentiating this category from the other disease patients, such as MSA and PSP. As a result, UCH-L1 may function as a diagnostic biomarker for PD with a satisfactory sensitivity of 89% and a moderate specificity of 67%. Moreover, there is a highly positive correlation between CSF UCH-L1 and CSF α-synuclein levels (Mondello et al., 2014). So, the combination of CSF UCH-L1 and α-synuclein levels are likely to become a more accurate diagnosis for prodromal PD.

β-Glucocerebrosidase, a lysosomal hydrolase encoded by *GBA1* gene, plays a key role in α-synuclein degradation (Sidransky et al., 2009). Lysosomal dysfunction and α-synuclein aggregation make great contributions to the pathogenesis of PD. Therefore, β-Glucocerebrosidase is recognized to be a potential risk factor for PD. In several studies, the activity of β-Glucocerebrosidase is confirmed to be declined in CSF of PD patients, especially in the early stage of PD (Hoehn-Yahr stage ≤2). Additionally, the combined determination of o-α-syn/t-α-syn ratio and β-Glucocerebrosidase activity will be of help in improving the accuracy of PD diagnosis with a sensitivity of 82% and a specificity of 71% (Parnetti et al., 2014).

#### Amyloid Beta 1–42 (Aβ42)

Amyloid Beta 1–42 (Aβ42), composed of 42 amino acids, is mainly derived from the hydrolysis of the amyloid precursor protein. As we all know, amyloid plaques resulting from the misfolding and polymerization of Aβ peptides are one of the classical pathological features of AD (Ballard et al., 2011). Furthermore, so many studies demonstrated a positive relationship between Aβ42 concentration and cognitive dysfunction. Decreased Aβ42 level makes contribution to the memory decline (Alves et al., 2010; Nutu et al., 2013). As a result, the reduced of Aβ42 in CSF plays a crucial role in early diagnosis of AD (Reijs et al., 2017). On the other hand, it also confirms that Aβ42 can contribute to the development of Lewy body diseases by promoting α-synuclein polymerization (Clinton et al., 2010). Although a large number of experiments suggested that the levels of CSF Aβ42 in PD patients were just a little lower than control subjects (Montine et al., 2010; Siderowf et al., 2010; Shi et al., 2011; Přikrylová Vranová et al., 2012). While CSF Aβ42 levels are significantly lower in AD patients, DLB patients or PD dementia (PDD) patients compared to normal controls and PD patients (Buongiorno et al., 2011; Vranová et al., 2014). To our surprise, Aβ42 levels are dynamically reduced from PD patients to cognitively impaired PD (CI-PD) patients to PDD patients who have the lowest concentrations. At the same time, PD patients who had developed into PDD at 18-months follow-up have been found to have obviously lower concentrations of CSF Aβ42 compared with the baseline levels. This result may be related to the pathogenesis of diffuse amyloid plaques and Lewy body pathology in neocortical regions in PDD patients (Mollenhauer et al., 2006; Montine et al., 2010; Andersson et al., 2011). It will provide powerful evidence for the utilization of Aβ42 in the assessment of cognitive abilities in PD patients with a sensitivity of more than 85% (Alves et al., 2014). Consequently, the reduced CSF Aβ42 levels are very likely to be one of the early biomarkers to accurately predict the development of PD patients from CI-PD to PDD.

#### Tau Protein

Tau, a microtubule-associated protein (MAP), is predominantly found in the axons of healthy neurons in both CNS and peripheral nervous system (PNS). It has been investigated to be an important pathological protein implicated in cognitive impairment. In AD patients, both total tau protein (t-tau) and phosphorylated tau protein (p-tau) levels are related to neuronal degeneration and tau tangle pathology, so raised levels of t-tau and p-tau in CSF are the diagnostic variations of AD (Constantinescu and Mondello, 2013). A large number of investigations showed the lower levels of t-tau and p-taus in CSF of PD compared with DLB, PDD, AD and MSA patients (Hall et al., 2012). PD patients can be distinguished from MSA by the combined CSF t-tau and p-tau levels with a sensitivity of 82% and a specificity of 81% (Herbert et al., 2014); and an inverse relationship between p-tau/t-tau ratio in CSF and the UPDRS or UPDRS motor score (UPDRS III) has been already confirmed (Zhang et al., 2013). Meanwhile, Parnetti et al. (2014) found that PDD patients have significantly higher CSF t-tau levels than that of PD patients without cognitive impairment (Andersson et al., 2011), and the PD patients who have higher concentrations of CSF t-tau and p-tau are more likely to suffer from impairment of memory and visuospatial dysfunction. In general, tau pathology seems to be associated with the cognitive impairment in PD patients, similar to AD patients. Tau protein may be used as an important predictor of cognitive decline in PD patients. Moreover, it was also suggested that the proportion of patients with a raised t-tau/Aβ index elevated from 15% in PD patients and 29% in CI-PD patients to 45% in PDD patients (Montine et al., 2010). So, when the tau protein is combined with Aβ, it will have more significantly prognostic value for predicting cognitive function in PD patients.

#### Neurofilament Light Chain Protein (NFL)

Neurofilaments, as the essential components of intermediate filaments in neurons, are the main structural components of axons in the PNS and CNS. The abnormal phosphorylation of neurofilaments has been detected in PD patients. As one of the three subunits, NFL plays a key role in the conduction of nerve impulses and maintenance of neuronal morphology integrity (Vågberg et al., 2015). It is a potential clinical biomarker of degeneration of large myelinated axons. However, there is no increase of NFL levels in neither CSF nor serum of PD patients (Hansson et al., 2017) due to the less severe axonal degeneration. But they increase in other neurodegenerative disorders such as PSP, MSA and CBD, et cetera (Constantinescu et al., 2010; Herbert et al., 2015; Magdalinou et al., 2015), reflecting axonal degeneration of large myelinated axons. Furthermore, NFL levels correlate with disease severity. The measurement of NFL in serum or CSF can be utilized to distinguish PD patients from PSP, MSA and CBS patients with an AUC of 0.81–0.91 (Hansson et al., 2017).

### Neurotrophins Related Biomarkers

#### Brain-Derived Neurotrophic Factor (BDNF)

Brain-derived neurotrophic factor (BDNF) is one of the most widely distributed neurotrophic factors that can regulate cell survival and synaptic plasticity as well as inhibiting apoptosis-mediated cell death (Scalzo et al., 2010a). Insufficient supply of neurotrophic factors will lead to the neurodegenerative process. In normal cases, BDNF is synthesized by neurons. But when neurons are injured, microglial cells can produce BDNF. Many studies suggested that the expression of BDNF in substantia nigra of PD patients is reduced, especially in ventral lateral (Howells et al., 2000). BDNF plays a significant role in supporting the survival of dopaminergic neurons. Consequently, recent research demonstrates that there are decreased BDNF levels in the serum of PD patients compared with normal control (Scalzo et al., 2010a). The lower BDNF levels in early stages of the disease may be of use for early diagnosis of PD, and an up-regulation of BDNF concentrations is likely to be a viable therapy for PD. However, there is a contradictory result that higher BDNF concentrations are associated with longer history of disease and more severe symptoms (Scalzo et al., 2010a). This paradox may result from the compensatory mechanism in the advanced stages of PD. Another research showed that the BDNF concentrations in the CSF of PD patients are much higher than those of control subjects due to the increased generation of glial cells resulting from brain damage (Salehi and Mashayekhi, 2009). Therefore, the combination of the BDNF concentrations in serum and CSF has become an early diagnostic marker for PD. On other hand, BDNF may regulate cognitive processes by regulating synaptic plasticity and activating signal transduction pathways. So there is a positive correlation between BDNF serum concentrations and cognitive performance (Costa et al., 2015). The measurement of BDNF in serum can be utilized to predict cognitive function as well as motor function.

#### Insulin-Like Growth Factor 1 (IGF-1)

Insulin-like growth factor 1 (IGF-1), secreted by the choroid plexus, exerts neuroprotective effect by inhibiting neuronal apoptosis and enhancing survival neurons (Fernandez and Torres-Alemán, 2012). It can reach neurons throughout the brain and act on the brain parenchyma. In normal case, neurons play a significant role in the synthesis of IGF-1. While in the damaged brain, reactive microglia will produce IGF-1. A large number of studies have demonstrated several correlations between IGF-1 and PD: (1) high densities of IGF-1 receptors exist in the substantia nigra; (2) IGF-1 increases the survival of dopaminergic and substantia nigra neurons; (3) IGF-1 protects dopaminergic neurons from programmed cell apoptosis (Mashayekhi et al., 2010; Bernhard et al., 2016). Several studies are consistent with these findings, showing that CSF IGF-1 levels are elevated in PD patients (Mashayekhi et al., 2010). At the same time, it has been demonstrated that serum IGF-1 concentrations in PD patients are higher than healthy controls (Godau et al., 2010, 2011). Further research suggests that only advanced stage PD patients (>3.5 years disease duration) have significantly higher levels of IGF-1 in serum than healthy controls. While early-stage PD patients (≤3.5 years disease duration) do not show a significant increase in serum IGF-1 levels (Bernhard et al., 2016). So, serum IGF-1 is regarded as a trait biomarker in advanced disease stages. These changes are caused by increased generation of microglia in PD patients because of brain destruction.

In fact, several IGF-1 receptors are reported in many brain regions crucial for cognitive performance, with the highest levels in the frontal cortex and the hippocampus. As a result, the decrease serum IGF-1 concentrations are associated with poor ability in execution and verbal memory in PD patients (Picillo et al., 2017). IGF-1 may become a helpful tool for the early detection of PDD.

### Neuroinflammatory Reaction Related Biomarkers

The neuroinflammatory response is significantly involved in the loss of dopaminergic neurons in PD patients (Mosley et al., 2012). It is confirmed in both PD patients and PD animal models that microglial cells in the nigrostriatal area are abnormally activated and involved in the inflammatory process. The activated microglia can secrete inflammatory cytokines such as IL-6, IL-1β and TNF-α to attack dopaminergic neurons and lead to the degeneration of neurons (More et al., 2013; Cebriã et al., 2014). At the same time, it can also present antigens to CD4^+^ T cells and CD8^+^ cytotoxic T lymphocytes cells by MHC-II pathway to take part in the immune regulation (Neefjes et al., 2011). Neuroinflammatory reactions will lead to the elevated secretion of IL-2, IL-6, IL-10, TNF-α, IL-1β and IFN-γ in plasm and CSF as well (Zhang et al., 2010; Scalzo et al., 2010b; More et al., 2013; Santiago and Potashkin, 2014). Therefore, the increase of these inflammatory factors may become a potential biomarker for the early diagnosis and detection of PD progression. On the other hand, it is also reported that elevated CSF IL-8 levels are prospectively related with a raised risk of cognitive impairment and decreased Montreal Cognitive Assessment (MoCA) scores in PD patients (Yu et al., 2014; Liu et al., 2016). Another study suggests that PDD patients have significantly higher mean concentrations of CSF C-reactive protein (CRP) than PD patients after controlling for confounders such as gender, age and somatic illness (Lindqvist et al., 2013). Therefore, IL-8 and CRP may be potential neuroinflammatory biomarkers for evaluating the severity of cognitive impairment in PD patients. In this case, medications targeting the inflammatory mediators may provide an effective treatment strategy for PD.

### Other Biomarkers

#### MicroRNA (miRNA)

The microRNA (miRNA), which were first described in 1993, are a group of single-strand, non-coding, small molecules that can regulate the expression of their target genes by messenger RNA degradation or translational inhibition (Khodadadian et al., 2018). Almost all PD-related genes are regulated by them, so these miRNAs have been involved in the pathogenesis of PD. MiRNAs expression typically vary depending on the progression of PD and the specific stage of the disease, resulting in the heterogeneity of the miRNAs. The dysfunction of these miRNAs can result in a series of problems, including downregulation of DJ-1 protein (Xiong et al., 2014; Zhang and Cheng, 2014), overexpression of α-synuclein (Zhang and Cheng, 2014), upregulation of pathogenic LRRK2 protein (Rassu et al., 2017), up- or down-regulation of inflammatory response (Nair and Ge, 2016; Zhou et al., 2016), dysregulation of the IGF (Kim et al., 2014), and even the death of dopaminergic neuronal (Chmielarz et al., 2017). Since circulating miRNAs are supposed to be tissue-specific, abundant, highly stable and quantifiable and they are up- or down-regulated several years before the onset of PD, a novel approach to using miRNAs as non-invasive biomarkers to detect the early PD and monitor the progression of the pathology has been proposed. As early as 2011, Martins and collaborators found 18 miRNAs were under-expressed in PD patients when carried a miRNA expression profiling study and they also identified that miR-26a, miR-30b and miR-30c were potentially correlated with PD susceptibility (Martins et al., 2011). Later on, Khoo et al. (2012) using microarrays for global miRNA expression analysis, they finally found four optimal candidate biomarkers: miR-450b-3p, miR-505, miR-626 and miR-1826. However, low predictive values were shown when they performed a further validation in different groups (Khoo et al., 2012). By using TaqMan low density array (TLDA) approach, (Cardo et al., 2013) found miR-331-5p upregulated in PD patients, and Vallelunga et al. observed three miRNAs (miR-24, miR-223* and miR-324-3p) upregulated and two (miR-30c and miR-148b) downregulated (Vallelunga et al., 2014). In 2016, Ding et al. identified five novel miRNAs by performing a RNA-seq approach, they found miR-195 upregulated and four miRNAs (miR-185, miR-15b, miR-221 and miR-181a) downregulated in PD patients (Ding et al., 2016; Leggio et al., 2017). Recently, 31 upregulated miRNAs and 19 downregulated miRNAs in PD patients were identified. Among those, miR-4639-5p was significantly upregulated. Importantly, it has nothing to do with gender, age of disease onset, severity of PD motor symptoms and L-DOPA treatment, making this miRNA a stable potential biomarker for the preclinical PD diagnosis (Chen et al., 2018). As we all known, miRNA profiling is not only promising to be a novel strategy for the diagnosis of PD, but also to provide new miRNA-based Therapies for PD treatment. Several strategies exist to modulate miRNAs levels inside cells. To target PD, exosomes may become natural and innovative transporters to deliver miRNAs into the brain across the blood–brain barrier.

#### Peptides

In the last few years, proteomic technologies have been used to identify new peptides correlated with different disease stages and severity. But there are no quantitative assays for the majority of these candidate peptides. Shi et al. (2011) were devoted to discovering novel biomarker through mass spectrometry (MS; Jaffe et al., 2008) and accurate inclusion mass screening (AIMS; Whiteaker et al., 2011) with high sensitivity, accuracy and specificity. They find that 17 peptides in CSF showed significant differences between PD and controls, and majority of these 17 peptides were independent of the age and gender of subjects. The specificity and sensitivity in PD diagnosis of these 17 peptides were evaluated using ROC analysis. Secreted phosphoprotein 1 (SPP1; AUC: 0.791; specificity: 56.7%; and sensitivity: 90.0%) and low density lipoprotein receptor-related protein 1 (LRP1; AUC: 0.706: specificity: 70.0%; and sensitivity: 70.0%) are supposed to be the best-performing individual peptides. Furthermore, a combination of five peptides, including SPP1, LRP1, Colony-stimulating factor receptor 1 (CSFR1), Ephrin type-A receptor 4 (EPHA4) and Tissue inhibitor of metalloproteinases-1 (TIMP1), can improve the AUC to 0.873 (specificity: 80.0%; and sensitivity: 76.7%) in differentiating PD patients from healthy subjects and can also differentiate PD from AD patients very well with an AUC of 0.990 (specificity: 97.4%; and sensitivity: 95.0%). This panel of peptides is very likely to be a biomarker that can provide good diagnostic sensitivity and specificity for PD as well as correlation with disease severity. These peptides play different roles in the occurrence and development of PD. Among these peptides, SPP1 is a glycosylated phosphoprotein expressed in neurons and appears to play the role of a double-edged sword in neurodegenerative diseases. It may be toxic to neurons and lead to cell death in some cases, but it also has powerful neuroprotective effects in others (Carecchio and Comi, 2011). LRP1 is a cell surface receptor implicated in signaling pathway. It can regulate clearance and transportation of Aβ and other ligands in the brain and maintain brain lipid homeostasis as well as neuronal integrity (Liu et al., 2010; Kanekiyo et al., 2013). CSFR1 is the receptor for colony stimulating factor 1 and IL-34 that can provide neuroprotective and survival signals in brain damage. The lack of CSFR1 can exacerbate excitotoxin-induced cell death and neurodegeneration (Luo et al., 2013). EPHA4, a member of the A subclass of Ephrin receptor tyrosine kinases, can guide axons during neural development and regulate neuronal plasticity and synapse formation through its ephrin ligands (Klein, 2009). TIMP1 functions primarily to inhibit a large class of matrix metalloproteinases (MMPs). It has been showed that MMP-3 contributes significantly to the pathogenesis of PD and other neurodegenerative diseases. First, MMP-3 is a key player in triggering neuroinflammation toward the pathophysiology of PD in response to neuronal cell stress. Second, MMP-3 can remove the negative charge of the C-terminal part of α-synuclein, making the C-terminally truncated protein highly hydrophobic prone to aggregation and much more cytotoxicity. In addition, DJ-1 that can provide protection against oxidative, proteasomal and mitochondrial stresses is also cleaved by MMP-3. Therefore, TIMP1 exerts neuroprotective effect in various ways (Kim and Hwang, 2011).

## Neuroimaging Biomarkers

Neuroimaging methodologies have become a mature biomarker for the nigrostriatal neurodegeneration by evaluating the potential structure, ultrastructural, or perfusion pattern changes in PD. An increasing number of neuroimaging technologies can be used to detect early changes in PD patients and assess progression of the disease, less susceptible to the effects of subjectivity and drugs.

### Transcranial Sonography (TCS)

Transcranial ultrasound is a new neuroimaging technique that can be utilized to directly observe midbrain structures and qualitatively evaluate their echogenic properties. This imaging technique can detect hyperechoic signals in the substantia nigra, which represents a dysfunction in the dopaminergic nigrostriatal pathway (Bouwmans et al., 2013). This change in the echogenicity of this signal is supposed to be associated with iron deposition that leads to the oxidative stress and thus further the impairment of dopaminergic neurons. So increased echogenicity is consistently found in more than 90% of PD patients. However, transcranial sonography (TCS) cannot accurately detect PD from other parkinsonian syndromes (Bouwmans et al., 2013), but it is a cheap, safe, non-invasive and painless technique. Consequently, TCS as a good neuroimaging biomarker for disease detection has become one of the PD diagnostic criteria for MDS 2015 (Postuma et al., 2015a).

### MRI

Although MRI is widely utilized in clinical practice in the differential diagnosis of parkinsonisms, routine imaging sequences can’t reliably detect specific neurodegeneration in PD patients. However, the capacity to identify related lesions has also been significantly improved with the emergence of several specific sequences and high field strength MRI scanners (Brooks and Tambasco, 2016). As we all know, the direct imaging technique of the substantia nigra has played an important role in the diagnosis of PD. Functional MRI (fMRI) can find out the disorder of connectivity network within the basal ganglion in PD patients by detecting the change of oxygenated hemoglobin and deoxyhemoglobin content in brain regions that varies with neuronal activity (Pyatigorskaya et al., 2014; Szewczyk-krolikowski et al., 2014). The changes of functional connectivity networks, which are related with propagation of abnormal aggregates of α-synuclein, can be revealed decades prior to any development of symptoms. So it maybe have potential to become a biomarker for preclinical PD (Tang et al., 2017). Furthermore, it has been reported that measurements of functional connectivity within the basal ganglia network based on the independent component analysis can detect PD patients from normal individuals with a sensitivity of 100% and a specificity of 89.5% (Szewczyk-krolikowski et al., 2014). Diffusion tensor imaging (DTI) which is utilized to assess the integrity of nigrostriatal fibers can detect a decreased fractional anisotropy in the substantia nigra of PD patients. However, there is a remarkable variation in results among DTI studies, so that we cannot accurately diagnose PD patients by using DTI (Schwarz et al., 2013). More promising, the visualization substantia nigra morphological changes in MRI may become a potential diagnostic neuroimaging biomarker for PD. The nigrosome-1, rich in melanin, situated in the caudal and medio-lateral substantia nigra of PD patients has the largest loss of neuromelanin containing dopaminergic neurons. Accordingly, it is found that characteristic low signals in the dorsolateral substantia nigra of PD patients are presented within the high field strength MRI with T2-weighted sequences because of the loss of neuromelanin containing dopaminergic neurons, while there are higher signals in normal controls (Blazejewska et al., 2013). On the other hand, the nigrosome-1, located in the third of the posterior substantia nigra, presents a linear, comma or wedge-shaped high signal on the SWI, while the medial lemniscus and the pars compacta situated in the anterior and lateral substantia nigra show low signals. Therefore, the normal nigrosome-1 and its surrounding structures were supposed to visually analog a “swallow-tail” on the axial SWI (Schwarz et al., 2014). As we all know, many studies suggest that iron, a free-radical involving in oxidative stress, plays an essential role in the pathogenesis of PD. An increasing number of iron deposition in the nigrosome-1 makes its signal getting lower and lower, and even the characteristic “swallow-tail” disappeared in PD patients (Blazejewska et al., 2013). The accuracy of diagnosis of PD by using this radiological assessment can be as high as 96% (Schwarz et al., 2014). The disappearance of typical “swallow-tail” on SWI is promising to be a new and easy applicable MRI diagnostic tool for PD with important clinical predictive value.

### Molecular Imaging Technology

To date, the molecular imaging technology has entered the new age of exploration into human brain diseases and its application has been widely used to support the clinical diagnosis of PD. Nowadays, the increasingly used molecular imaging techniques focus on both dopaminergic and non-dopaminergic imaging.

#### Dopaminergic Imaging

The pathologic changes in PD patients mainly occur in the substantial nigrostriatum, with the gradual degeneration of dopaminergic neurons. Although dopamine levels cannot be measured directly by using imaging, various methods can be used to assess altered function of nigrostriatal dopaminergic neurons terminals. The most easily accessible approach is the use of markers for the dopamine transporter (DAT). Functional cerebrum imaging using tracers that can penetrate the blood-brain barrier can identify diseased areas in the cerebrum with either positron emission tomography (PET) or single photon emission computed tomography (SPECT). At the same time, there are three types of radiotracers commonly utilized in the DAT, including 6-[18F]-fluoro-L-3,4-dihydroxyphenylalanine (18F-DOPA), dihydrotetrabenazine (DTBZ) and 2-beta-[11C]-carbomethoxy-3-beta-4fluorophenyltropane (11C-CFT).

18F-DOPA is a fluorinated form of levodopa (L-DOPA), extensively utilized as a radiotracer in the PET scans. This scan imaging is the first neuroimaging technology used to evaluate the integrity of presynaptic dopaminergic neurons. When 18F-DOPA is injected into the blood, it penetrates the blood-brain barrier, and then it will be taken up by axonal terminals of dopaminergic neurons. Ultimately it converts into fluorodopamine (18F-DA) by decarboxylation of enzyme aromatic amino acid decarboxylase (AADC). The uptake of 18F-DOPA by the striatum, which reflects the density of the axonal terminal plexus and the activity of AADC, is positively related with the remaining substantia nigra dopaminergic cell counts correlating well with remaining nigral dopaminergic cell counts. Using serial 18F-DOPA PET, a prospective study has found obvious declines in 18F-DOPA uptake in ventral anterior thalamus and putamen of early-stage PD patients at baseline compared to normal controls, while there is a significant decrease in globus pallidus internal and raphe of advanced PD patients compared with both early-stage PD patients and control subjects (Pavese et al., 2009). This cause may be that 18F-DOPA PET is likely to underrate the degree of the neurologic degenerative process in the early stages, because of the compensatory upregulation of AADC in remaining functional terminals (Moore et al., 2008). As a result, its utility in detecting early stage of the disease maybe questionable, but 18F-DOPA PET is likely to be available in identifying symptomatic PD patients.

DTBZ and 11C-CFT (DAT biomarker) which are related with striatal dopamine and fiber density are reflections of terminal field function. Vesicular monoamine transporter 2 (VMAT2), which is a membrane protein, is responsible for packaging monoamines from the cytosol into their appropriate secretory synaptic vesicles. VMAT2 is typically imaged by using DTBZ PET and is correlated well with total counts of nigral dopaminergic neuron bodies. Decreased DTBZ uptake reflects nigrostriatal degeneration. Multiple studies reveal that the threshold for PD clinical manifestations is 29% to 44% of normal values for 11C-CFT uptake and 38% to 49% for DTBZ uptake in early stage of PD patients (Lee et al., 2000). Moreover, it is confirmed that the striatal DTBZ is apparently decreased with the putamen showing the largest reduction in PD patients. So their uptake decreases with disease progression and negatively correlates with clinical disease severity (Bohnen et al., 2006). In fact, almost all of these presynaptic dopaminergic markers show a very analogous pattern in PD patients, with asymmetric involvement of the striatum, and a rostral-caudal gradient in which the posterior putamen is greatest affected and the caudate nucleus least (Nandhagopal et al., 2009). PET measures of uptake of VMAT2 or DAT become promising biomarkers for evaluating the severity of nigrostriatal injury. As a result, normal functional imaging of the presynaptic dopaminergic system has become one of the PD absolute exclusion criteria for MDS 2015.

On the other hand, it has been reported that dopamine release is also likely to be an effective method to assess dopaminergic functions. This can be evaluated by making use of the relatively high affinity of several tracers for postsynaptic D2/D3 dopaminergic receptors. We can examine the postsynaptic dopaminergic receptors by the PET ligand 11C-raclopride for D2/3 receptor or SPECT tracer 123I-iodobenzamide (123I-IBZM) for D2 receptors (Plotkin et al., 2005; Derlin et al., 2010). Verstappen et al. (2007) suggested that there is an upregulation of the D2 receptors resulting from progression of dopamine depletion in untreated PD patients. However, there is a significantly decrease in 11C-raclopride binding in PSP patients and MSA patients compared with PD patients and normal controls (Ghaemi et al., 2002; Kashihara et al., 2011). Especially, mean 11C-raclopride local influx ratios in posterior putamen, bilateral cerebellum and bilateral pons of the PD patients are significantly higher than other Parkinsonian syndromes (Van Laere et al., 2010). IBZM SPECT can distinguish PD patients from L-DOPA non-responsive Parkinsonian syndromes with an accuracy of 87%, a sensitivity of 62% and a specificity of 100% (Plotkin et al., 2005). It seems that the negative predictive value of the PD diagnosis is low, only 62%. Therefore, the negative results of IBZM scan can’t rule out atypical PD patients and they need for follow-up observation. By contrast, a positive finding of IBZM SPECT is pretty specific for the diagnosis of atypical PD because the positive predictive value is 100%. To sum up, it is appropriate to use postsynaptic tracers to diagnose early and untreated PD.

#### Non-dopaminergic Imaging

Multiple studies suggested that nondopaminergic mechanisms may play a key role in the pathophysiology of PD. Experiments carried out with serotonergic PET tracers have revealed the effect of this neurotransmitter.

123I-MIBG, can be taken up by the postganglionic adrenergic neurons, is a radioiodinated analog of guanethidine. The uptake and localization of 123I-MIBG provide an available indicator of integrity and function of postganglionic sympathetic fiber. Uptake of 123I-MIBG in myocardial scintigraphy has been widely used to evaluate sympathetic nerve impairment in cardiovascular diseases by using a heart-to-mediastinum (H/M) ratio of count densities. In recent years, 123I-MIBG also can be utilized in the differential diagnosis of neurodegenerative diseases, particularly in α-synucleinopathies because we can find severe cardiovascular dysautonomia in these diseases (Orimo et al., 2016). Extensive studies have found an obvious decrease of 123IMIBG uptakes in PD patients compared with normal controls, even at the preclinical PD patients lack of clinically apparent dysautonomia (Oka et al., 2011; Kaufmann and Goldstein, 2013). However, uptake of 123I-MIBG in PSP, MSA and CBS patients is basically normal or mildly decreased. So 123I-MIBG SPECT will assistant differentiate PD patients from other parkinsonian syndromes, with a sensitivity of 88% and a specificity of 85% (Treglia et al., 2012; Yoshita, 2014). Glucose metabolism imaging plays an increasingly significant role in identifying parkinsonian syndromes. In PD patients, 18F-FDG-PET usually shows comparatively preserved glucose metabolism in the thalamus and lentiform nucleus (Zhao et al., 2012; Akdemir et al., 2014), but hypometabolism in the premotor and supplementary motor regions and bilateral parietal compared with normal controls (Poston and Eidelberg, 2010; Zhao et al., 2012). PD patients who exhibit impaired metabolism in the basal ganglia can be discriminated from PSP and MSA patients by the preserved glucose metabolism in bilateral basal ganglia (Zhao et al., 2012; Akdemir et al., 2014). Furthermore, Eidelberg et al. identified a PD-related pattern (PDRP) by quantitative analysis of FDG-PET data (Eidelberg, 2009). PDRP, which is characterized by raised pontine and pallid-thalamic metabolic activity with comparatively decrease in premotor cortex, supplementary motor area and parietal association regions (Eidelberg, 2009), reveals linear relationship with motor assessments, and can distinguish PD patients from other parkinsonian syndromes such as MSA and PSP (Tang et al., 2010).

## Genetic Biomarkers

The cause of PD has not been completely understood so far, but the complex interactions between genes and environmental factors may be involved (Delenclos et al., 2016). As we all know, it has been reported that the risk of suffering from PD in individuals having a family history is 3.0–4.5 times that of people without it (Schulte and Gasser, 2011; Noyce et al., 2012). Genetics are considered to have an important influence on susceptibility to PD. Up to date, at least 20 genes have been confirmed to be linked to familial forms of PD, while genome wide association studies (GWAS) have identified more than 23 PD genetic risk loci (Deng et al., 2017). From these, eight genes are supposed to be related with autosomal-recessive modes of inheritance, of which *Parkin*, *DJ-1* and *PINK1* are associated with typical early-onset PD, and *ATP13A2*, *DNAJC6*, *PLA2G6*, *FBOX7* and *SYNJ1* are correlated with atypical forms of juvenile-onset PD. The other three genes including *SNCA*, *LRRK2* and *VPS35* are found to lead to typical autosomal dominant PD (Guo et al., 2015; Shen et al., 2016); and analysis for genetic mutations in *Parkin*, *DJ-1*, *PINK1*, *SNCA*, *LRRK2* and *GBA* are much of importance, which account for 2%–3% of PD patients. For instance, the point mutations, duplications and triplication in the *SNCA* gene will lead to high penetrance in PD patients. *SNCA* encodes the α-synuclein, the principal component of Lewy bodies, and mutations of this gene accounts for more than 1% in the general population (Siddiqui et al., 2016). Additionally, 5%–10% of PD patients have been reported to have mutations in *GBA*, so the *GBA* gene has become the most significant genetic risk factor for PD until now (Beavan et al., 2015). Genetic factors of the etiology of PD may maintain in patients for decades before onset of clinical disease. Consequently, the positive genes in peripheral blood, like the proteins related with disease pathophysiology, is likely to becoming candidate biomarkers for the diagnosis of PD and parkinsonian syndromes (Yang et al., 2015).

## Prospect

A large number of research groups have been exploring the new approaches to discover PD biomarkers. So far, they have found numerous candidate biomarkers. Biomarkers, as indicators of normal physiological or pathological processes, are always involved in disease mechanisms. Given the complexity of the disease etiology and pathological cascades, a more comprehensive identification of specific disease networks and molecular pathways will help us better understand the disease process and find more effective biomarkers. In recent years, the -omics tools, such as transcriptomics, proteomics and metabolomics, have been already in place and have been implemented in the study of PD biomarkers. Transcriptomics is primarily concerned with the identification of genes and gene expression. It is suggested that the high-throughput capabilities of gene microarrays and the relative availability of blood components from PD patients and healthy controls can be used to get a more focused understanding of the role of specific proteins in PD pathogenesis and to identify potential biomarkers of PD (Caudle et al., 2010). Proteomics is a discipline focusing on the study of protein structure and function. It consists of several integrated technical components, including separation technology, MS and bioinformatics data processing. Proteomics can provide a high-throughput platform and generate massive data about the identification of proteins and quantification of their relative changes in expression from one disease state to another. Proteomics will provide a more comprehensive and in-depth understanding of the underlying pathogenesis of PD to find unique protein markers through the use of different body tissues, such as plasma, serum, CSF or tissue (Misato et al., 2014). Metabolomics focuses on the identification and quantification of metabolites to provide a means of differentiating PD patients from normal controls, regardless of medication status. Metabolomic analysis using electrochemical coulometric array detection has the potential for discovery of metabolic profiles that can be used as novel biomarkers. Lewitt previously identified 19 compounds that can differentiate PD patients from controls by using this technique (Amara and Standaert, 2013). Consequently, the -omics tools are promising to become new approaches to discovering sensitive PD biomarkers.

Many promising biomarkers have been proposed so far, but the ideal biomarkers are still elusive. As we all know, biomarkers always play different roles at different stages of the disease process. In addition, the various biomarkers profiles vary widely from person to person. A single biomarker may not be sufficient to diagnose diseases early and predict disease course with adequate sensitivity or specificity. It is necessary to develop combination method to identify prodromal PD using comprehensive biomarkers dataset consisting of clinical characteristics, biospecimen, neuroimaging and genetic information. The recent works of several teams provided many examples of using a combination of multimodal biomarkers in PD patients. In 2015, Nalls et al. designed an accurate, non-invasive integrative predictive model to identify preclinical and prodromal PD patients. In comparison of the standardized β coefficients of this regression model, the UPSIT score was responsible for 63.1% of the explained variance, the genetic risk score for 13.6%, family history for11.4%, gender for 6.0%, and age for 5.9% (Nalls et al., 2015). In 2017, Latourelle et al. developed predictive models using clinical information, molecular biomarkers, imaging variables and genetic data to better predict disease progression in PD patients. Finally, 12 important predictors were identified to detect those patients who are at risk of rapid disease motor progression. They are well predictive accuracy in both early and late stages of the disease (Latourelle et al., 2017). In addition, Schrag et al. (2017) utilized several clinical information and biomarker variables as predictive factors to identify people at risk of cognitive decline. They suggest that the combination of UPSIT score, RBD Screening Questionnaire (RBDSQ), CSF Aβ42, and mean caudate uptake on DAT imaging can predict cognitive impairment at 2 years with an AUC of 0.80 (Schrag et al., 2017). Fereshtehnejad et al. (2017) also developed an approach for identifying distinct PD subtypes via cluster analysis of a comprehensive list of these various biomarkers to predict progression with a satisfactory accuracy.

## Conclusion

**Figure 1:**
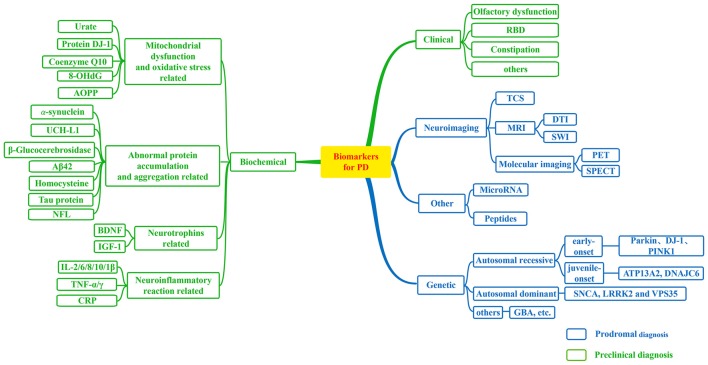
Biomarkers for Parkinson’s disease (PD). A flow figure illustrates various major biomarkers in PD. Biomarkers can be divided into following broad categories; and biomarkers in blue grids can be used to prodromal diagnosis for PD, while biomarkers in green grids may be helpful in the detection of preclinical PD as illustrated in this diagram.

Up to now, research on biomarkers for PD has made significant progress and an increasing number of candidate biomarkers for PD have been found (Figure 1). These biomarkers will make great contributions to the diagnosis or differential diagnosis of PD. So that we can make an early intervention in the disease and delay the progress of the disease. With limited ability to diagnose PD by a single biomarker, we should make more efforts to joint different biomarkers to improve the diagnostic accuracy of PD with higher sensitivity and specificity. In future, comprehensive and combined biomarkers are likely to be widely utilized in the safe and effective diagnosis and treatment of PD. In addition, further studies of biomarkers will provide new avenues for disease treatment, especially at early stage of PD.

## Author Contributions

QS and RH discussed the concepts and wrote the manuscript. XY, JG, QX and BT reviewed the literature and revised the manuscript.

## Conflict of Interest Statement

The authors declare that the research was conducted in the absence of any commercial or financial relationships that could be construed as a potential conflict of interest.
